# Tracking of In-111-labeled human umbilical tissue-derived cells (hUTC) in a rat model of cerebral ischemia using SPECT imaging

**DOI:** 10.1186/1471-2342-12-33

**Published:** 2012-12-06

**Authors:** Ali S Arbab, Christine Thiffault, Bradford Navia, Stephen J Victor, Klaudyne Hong, Li Zhang, Quan Jiang, Nadimpalli RS Varma, ASM Iskander, Michael Chopp

**Affiliations:** 1 Department of Radiology, Cellular and Molecular Imaging Laboratory, Henry Ford Hospital, Detroit, MI 48202, USA; 2 Department of Radiology, Wayne State University School of Medicine, Detroit, MI, USA; 3 Advanced Technologies and Regenerative Medicine, LLC, a Johnson & Johnson Co., Route 22 West, P.O. Box 151, Somerville, NJ 08876-0151, USA; 4 Eisai, Inc, 155 Tice Blvd, Woodcliff Lake, NJ 07677, USA; 5 Department of Neurology, Henry Ford Hospital, Detroit, MI, USA; 6 Department of Physics, Oakland University, Rochester, MI, USA

**Keywords:** Human umbilical tissue-derived cells (hUTC), In-111-oxine, SPECT, Cell tracking, Stroke rats

## Abstract

**Background:**

In order to increase understanding of how infused cells work, it becomes important to track their initial movement, localization, and engraftment efficiency following transplantation. However, the available *in vivo* cell tracking techniques are suboptimal. The study objective was to determine the biodistribution of intravenously administered Indium-111 (In-111) oxine labeled human umbilical tissue-derived cells (hUTC) in a rat model of transient middle cerebral occlusion (tMCAo) using single photon emission computed tomography (SPECT).

**Methods:**

Rats received 3 million In-111 labeled hUTC (i.v.) 48 hrs after tMCAo. Following the administration of either hUTC or equivalent dose of In-111-oxine (18.5 MBq), animals underwent SPECT imaging on days 0, 1, and 3. Radioactivity in various organs as well as in the stroke area and contralateral hemisphere was determined, decay corrected and normalized to the total (whole body including head) radioactivity on day 0. Immunohistochemical analysis was also performed to confirm the beneficial effects of hUTC on vascular and synaptic density, and apoptosis.

**Results:**

Most of the radioactivity (43.36±23.07% on day 0) trafficked to the lungs immediately following IV administration of In-111 labeled hUTC (day 0) and decreased drastically to 8.81±7.75 and 4.01±4.52% on days 1 and 3 post-injection, respectively. In contrast, radioactivity measured in the lung of animals that received In-111-oxine alone remained relatively unchanged from day 0 to day 1 (18.38±5.45% at day 0 to 12.59±5.94%) and decreased to 8.34±4.25% on day 3. Significantly higher radioactivity was observed in stroke areas of animals that received In-111 labeled hUTC indicating the presence of cells at the site of injury representing approximately 1% of total administered dose. In addition, there was significant increase in vascular and synaptophysin immunoreactivity in stroke areas of rats that received In-111 labeled hUTC.

**Conclusions:**

The present studies showed the tracking of In-111 labeled hUTC to the sites of stroke in a rat model of tMCAo using SPECT. Animals treated with In-111 labeled hUTC showed histological improvements, with higher vascular and synaptic densities observed in the ischemic boundary zone (IBZ).

## Background

On average, every 40 seconds someone in the United States has a stroke [1]. Each year, about 795,000 people in the United States experience a new or recurrent stroke. About 610,000 of these are first attacks, and 185,000 are recurrent attacks. Stroke mortality in 2008 accounted for about 1 of every 18 deaths in the United States. Of all strokes, 87% are ischemic, 10% are intracerebral hemorrhagic, and 3% are subarachnoid hemorrhagic strokes [2].

The current treatment for ischemic stroke, tissue plasminogen activator (TPA), while efficacious, is only effective if it is administered within 4.5 hours of the ischemic event [3,4]. Therefore, treatment with TPA remains inadequate for patients who are unable to be diagnosed and treated within that time frame. TPA is also not appropriate for patients who suffer a hemorrhagic stroke. Considering the limitations to TPA and the high mortality rate of stroke patients, there is a clear, unmet, need to reduce mortality rates, restore neural function and extend the current window of therapeutic opportunity. Effective treatments will have to have a wide window of therapeutic opportunity, address the neurological damage associated with stroke and reduce disabilities that severely reduce quality of life for stroke patients. Cell-based therapy, a novel approach to treating stroke, may offer significant health benefits to patients [5-7].

Umbilical tissues and cord blood are sources of stem cells. Cells from human umbilical cord blood (HUCBC) have been extensively characterized [8-10] and most show hematopoietic markers, such as CD34 and CD45. Unlike human umbilical cord blood derived stem cells, we have used human umbilical tissue-derived cells (hUTC), which are CD34 and CD45 negative cells. The detailed procedures of collection, separation and characterization have been reported by others [11]. hUTC are identified as a potential technology for neurorestorative and to improve functional outcomes following stroke. These cells have been characterized with respect to the secretion of trophic, proangiogenic and neuroprotective factors that could address the cellular and structural damage to the brain post-stroke [11,12]. hUTC have recently been shown to be efficacious in animal models of diseases and an ability to improve neurological functional recovery in animal models of cerebral ischemia [12]. hUTC may modulate restorative processes including vascularization, and synaptic plasticity [1,12].

In order to increase understanding of how infused cells work, it becomes important to track their initial movement, localization, and engraftment efficiency following transplantation. However, the available *in vivo* cell tracking techniques are suboptimal. For instance, *in vivo* fluorescent or bioluminescent molecular and/or cellular imaging techniques lack the resolution necessary to localize sites of active cell migration and accumulation. Superparamagnetic iron oxide (SPIO)-transfection agent complexes using two FDA approved agents, ferumoxides (Fe) and Protamine sulfate (Pro) have been created to label a broad range of mammalian cells. The labeled cells can then be used as probes to localize physiological or pathological processes using magnetic resonance imaging (MRI) for high-resolution images in clinical setting [13,14]. Cells labeled with the ferumoxides-protamine sulfate (FePro) complexes can be imaged at clinically relevant MRI fields using standard imaging techniques and also at higher fields typical for animal experiments. However, current MRI methods can not differentiate focal hemorrhage from accumulated iron positive cells. Many physiological and pathological conditions like hemorrhage causes similar T2* effects on MRI signal as iron containing contrast agents, and therefore cause possible misinterpretation of iron containing contrast agent accumulation. On the other hand, cells labeled with radioisotopes such as In-111-oxine can be tracked with confidence due to higher sensitivity of nuclear medicine imaging modalities and availability of hardware and software for whole body scanning. Due to minimal background signal, accumulation of In-111-labeled cells can also be quantified. However, quantitative estimation of labeled cell concentration by MRI is under development and could produce significant errors due to large background signal from subject interfaces and pathological conditions (hemorrhage etc.). These errors could be more significant in the body than that in the brain.

The purposes of this study were to determine, whether an *in vivo* imaging modality such as single photon emission computed tomography (SPECT) can be applied to determine the migration and localization of In-111- labeled hUTC to the sites of stroke in a rat temporal middle cerebral artery occlusion (MCAo) stroke model and to determine the biodistribution of administered In-111 labeled hUTC to various organs over time. For the future prospect of clinical trials using hUTC, it is important to know the whole body bio-distribution, organs of initial homing and dynamics of redistribution, and specific accumulation profiles following systemic administration.

## Materials and methods

### Ethics statement

Animal experiments described in the manuscript were approved by the animal care and user committee at Henry Ford Health System according to the guidelines and policies of office of laboratory animal welfare (OLAW) and public health service, National Institutes of Health. All the experiments were performed according to the approved protocol. Human umbilical tissue-derived cells (hUTC) were isolated and expanded from the umbilical cord of a single donor under full written consent, as approved by the institutional review board (IRB) of Lonza Inc (Allendale, NJ, USA) according to USA FDA requirements. All consent forms are protected as described in the approved IRB protocol.

### Collection and preservation of Human Umbilical Tissue Derived Cells (hUTC)

Human umbilical tissue-derived cells (hUTC) were isolated and expanded from the umbilical cord of a single donor under full consent, as previously described [11,12]. These cells are positive for CD10, CD13, CD44, CD73, CD90 and HLA-ABC (MHC I) but negative for CD31, CD34, CD45, CD117 and HLA-DR, -DP and –DQ (MHC II). Aliquots of the cell bank were thawed for characterization testing that included viability, recovery, sterility, endotoxin, mycoplasma, karyology and cell surface marker immunophenotyping to ensure safety and identity. hUTC were also tested at an early passage for viral pathogens by a PCR-based method. Cells were stored at cryogenic temperatures until labeling with In-111 oxine.

### Animal model

Seven to eight week-old male Wistar rats weighing 270–300 g were purchased from Charles River Laboratories International (Wilmington MA). Total of 25 animals were included in this study. The animals utilized in this study were handled and maintained according to protocols and guidelines approved by the Institutional Animal Care and Use Committee at Henry Ford Health System in accordance with the current requirements of the Animal Welfare Act (9 CFR) and the current standards set forth in the Guide for the Care and Use of Laboratory Animals. Temporal middle cerebral artery occlusion (tMCAo) was induced in rats using the modified method of intraluminal vascular occlusion as previously described [12]. Briefly, rats were anesthetized with 3.5% isoflurane and maintained with 1-2% isoflurane in 70% N_2_O and 30% O_2_ using a face mask. The right common carotid, external carotid (ECA) and internal carotid (ICA) arteries were exposed. A length of 4.0 monofilament nylon suture (~19 mm) with its tip rounded by heating near a flame was introduced from the ECA into the lumen of ICA until it blocked the origin of the middle cerebral artery (MCA). Two hours after MCA occlusion, animals were re-anesthetized with isoflurane and reperfusion was achieved by withdrawal of the suture until the tip cleared the lumen of the ICA.

### Preparation of In-111 labeled hUTC and In-111 oxine

Based on an initial labeling and In-111 cellular retention study results (supporting information S1), cells were labeled under adherent conditions. Cryopreserved cells were thawed in a 37°C water bath, washed and resuspended in DMEM-low glucose media containing 15% (vol/vol) FBS (HyClone, Logan UT), 100U/ml penicillin and 100 μg/ml streptomycin (Invitrogen, Carlsbad, CA). Six million cells were plated onto a T75 flask and culture for 48 hrs. On the day of In-111 labeling, the cells were washed twice with 5 ml of DMEM (serum free) and 2 ml of DMEM (serum free) was added to the flask. Thirty seven MBq of In-111 oxine (in 1 ml in PBS, Anazao Health Corp, Tampa FL) was added; adherent cells were gently rocked and incubated at 37°C for 20–25 min. The supernatant was removed and kept aside to measure labeling efficiency (see below). The cells were washed twice with PBS containing Ca^2+^/Mg^2+^ and the collected supernatants were combined with the kept aside supernatant. Cells were detached using TrypLE (Invitrogen Corp, Carlsbad CA) and 7 ml DMEM containing 20% FBS was added to neutralize TrypLE. Cells were centrifuged 150 × g for 5 minutes at room temperature and the supernatant was combined with the previous washes (labeling efficiency; see below). Cells were resuspended in 2 ml of a proprietary serum-free cell delivery formulation, and the radioactivity content was measured (see below). Cell viability was determined using the Trypan blue exclusion method and viability of 80% or greater was used in this study. The radioactivity content in the cell suspension and the combined washes were counted using a dose calibrator (CRC 127R, Capintec Inc., Ramsey NJ). Labeling efficiency was approximately 55%. Rats were administered (i.v. tail vein) either 3 million cells labeled with 18.5 MBq in a 2 ml volume or vehicle (18.5 MBq Indium-111 oxine) 48 hrs post-tMCAo. The number of administered cells was selected based on our previous study [12]. Labeling efficiency was calculated as follows:

% labeling efficiency or cell associated radioactivity = (radioactivity in cell suspension) / (radioactivity in cell suspension + total radioactivity in all supernatant and cell washes)*100

### Image acquisition

Animals were randomly assigned into two groups; 1) animals receiving an intravenous (tail vein) administration of In-111-labeled hUTC 48 hrs post-tMCAo (n=13), and 2) animals receiving an equal amount of In-111 oxine radioactivity 48 hrs post-tMCAo (n=12). Following the administration of either In-111-labeled hUTC or In-111-oxine alone, all animals underwent SPECT on days 0 (at least 1 hour after injection, 1–3 hours), 1 and 3 post administrations. Following anesthesia all animals underwent SPECT imaging with a dedicated PRISM 3000 gamma camera fitted with custom built multi-pinhole rat collimator (Bioscan, Washington DC), 360 degree rotation with 36 degree increments, 180 sec per projection, using 256x256 matrices with a field of view of 4x6 cm. Both whole body and head only images were acquired. Following acquisition, all images were transferred to a separate computer for reconstruction and image analysis. Reconstruction was performed by dedicated software supplied by Bioscan (HiSPECT, Washington DC). Image analysis was performed using Image J software (NIH, Bethesda MD). A total of 25 animals received either In-111-oxine alone (12 animals) or In-111-labeled hUTC (13 animals). Animals in the vehicle and treatment groups received an equivalent dose of 18.5 MBq of In-111. Maintaining the acquisition time 180 sec per projection, some animals underwent SPECT acquisition on day 7, however a reliable 3D reconstruction was not possible due to In-111 physical/biological decay. Therefore, we have not included images from day 7.

### Image analysis

**Figure 1:**
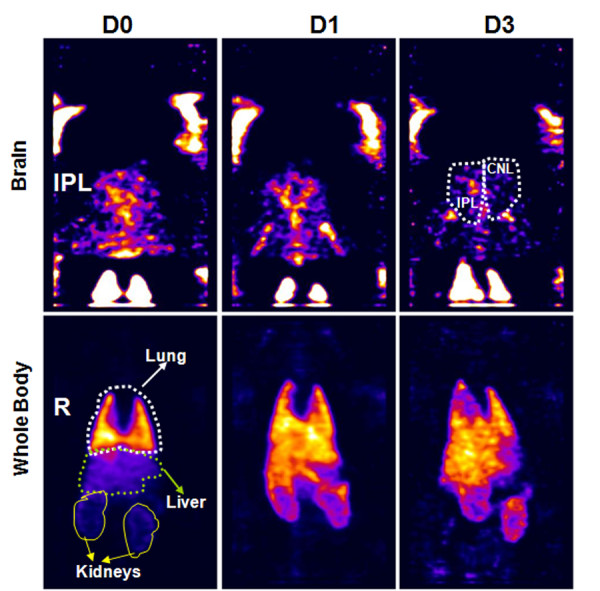
**Representative images of an animal administered 3 million In-111 labeled hUTC.** Most of the cells trafficked to the lungs soon after administration (day 0) and gradually decreased over time with increasing radioactivity observed in the liver. Note the early accumulation of radioactivity in ipsilateral (right side) of the brain (tMCAo side) on day 0. IPL = ipsilateral, CNL = contralateral, R= right side.

**Figure 2:**
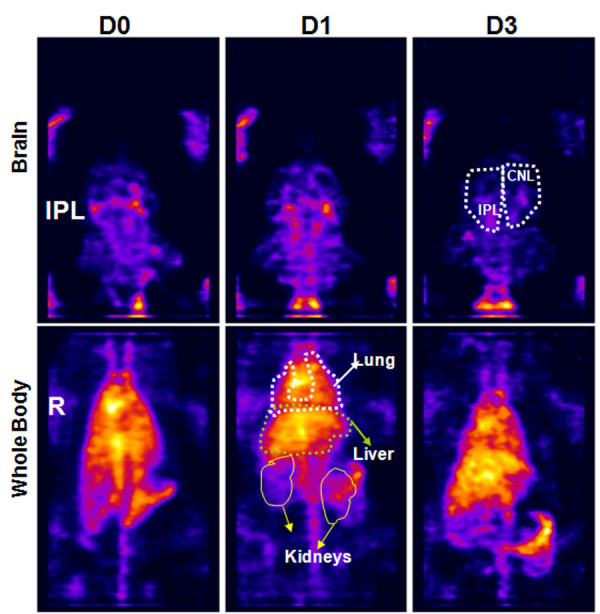
**Representative images of an animal treated with In-111 oxine alone (vehicle).** The radioactivity observed in the lung and liver remained relatively constant over time. Note that there was no preferential increase in the radioactivity in the ipsilateral (right) brain (tMCAo side). IPL = ipsilateral, CNL = contralateral, R = right side.

Multiplanar reconstruction was performed using a slice thickness of 0.8 mm. Volumetric images were created by adding slices from the entire animal, dorsal to ventral. Volumetric images of the ipsilateral (site of tMCAo) and contralateral hemispheres were created by adding slices of the brain but excluding the slices of bones and base of the skull. However, to determine whole body radioactivity, all slices of the head including bones, base of the skull and brain were added and the radioactivity was determined. Radioactivity in the animal (whole body including head) on day 0 following administration of either In-111-labeled hUTC or In-111-oxine alone (vehicle) was considered the total administered dose. Regions of interest (ROIs) were drawn on the ipsilateral (site of lesion) and contralateral intact brain excluding high radioactivity measured in the petrous bones, lungs, liver, spleen and both kidneys (Figures 1 and 2). Measured radioactivity in various organs was reported as the percent of the whole body radioactivity representing the administered dose (decay corrected for In-111 half-life). Percent administered dose was calculated for the lungs (excluding heart radioactivity), liver, spleen and kidneys on days 0, 1 and 3. Whole body radioactivity was also determined for all animals on days 0, 1 and 3 following administration of In-111-labeled hUTC and In-111-oxine. The ROIs were created by an investigator who is well versed in the anatomy of animals depicted on nuclear medicine studies. Although co-registration was not possible due to unavailability of concurrent whole body anatomical images, the investigator was careful enough to encircle only the organ of interest.

### Neurobehavioral evaluation

All animals that received either 3 million In-111 labeled hUTC or In-111-oxine only 48 hours following tMCAo underwent neurobehavioral evaluation such as; modified neurological severity score (mNSS), adhesive removal test, and foot-fault test at pre, day 1, day 14, and weekly thereafter for up to 90 days after tMCAo as previously described [15-17]. Detail procedures of these tests have been described in our recent publication [18].

### Brain section preparation and evaluation

To determine the infarct volume, vascular and synaptic densities at the sites of the lesion, animals were euthanatized at 12 weeks after the initiation of tMCAo. Following euthanasia, rats were perfused with saline and 4% paraformaldehyde. Then the brain was collected and immersed in 4% paraformaldehyde. Seven 2 mm thick paraffin blocks were made from each rat brain for histochemical analysis.

### Measurement of infarct area

One of each coronal paraffin section (6 μm thick) from seven blocks was stained with hematoxylin and eosin (H&E). The seven brain sections were traced using the MCID computer imaging analysis system (InterFocus Imaging Ltd, Linton, Cambridge). The lesion area was measured indirectly by subtracting the intact area of the ipsilateral hemisphere from the area of the contralateral hemisphere [19]. The lesion volume is presented as a volume percentage of the lesion compared to the contralateral hemisphere.

### Vascularization and synaptic density

A paraffin block was obtained from the center of the lesion, corresponding to coronal coordinates for bregma −1~1 mm. A series of 6-μm-thick sections were cut from this block for analysis by light microscopy (Olympus model BH-2, Olympus America Inc., Center Valley PA). To determine the vascular and synaptic densities around the stroke areas in In-111-labeled hUTC and In-111 oxine treated groups, antibodies against vonWillebrand factor (vWF, 1:400, Dako, Carpinteria CA) and synaptophysin (1:500, Chemicon, Billerica MA) were used to identify vessels and synapses, respectively.

### Apoptotic cell staining

The terminal deoxynucleotidyl transferase (TdT)–mediated dUTP-biotin nick end labeling (TUNEL) method (in situ Apoptosis Detection Kit, Chemicon, Billerica MA) was used to identify and assess apoptotic cells in situ. The TUNEL method is based on the specific binding of TdT to 3’-OH ends of DNA and the ensuing synthesis of polydeoxynucleotide polymer cells. Staining was performed according to procedures provided by the manufacturer.

### Presence of human cells and host macrophages

To confirm the migration and distribution of administered hUTC in and around the stroke lesion, especially in ischemic boundary zone (IBZ), sections were also stained with human cell specific marker such as β2-mitochondria (anti human β2-mitochondria antibody, Chemicon, Billerica MA). Human cell specific mitochondrial antibodies are described in previous publications [20]. The antibody that we used is also specific for human mitochondria and does not react with rat or mouse ( http://www.millipore.com/catalogue/item/mab1273) [21,22]. To determine the presence of host macrophages at the sites of stroke and their presence around the accumulated human specific cells, sections were double stained with anti human β2-mitochondria antibody (secondary antibody tagged with Rhodamine) and anti rat macrophage antibody (CD68, Santa Cruz, secondary antibody tagged with FITC) and counter stained with DAPI.

### Apoptotic cell, vascular and synaptic density quantification

The number of apoptotic cells per section was counted in the ipsilateral hemisphere. For measurement of vascular and synapse density, 8 fields of view from the IBZ were digitized using a 40X objective and the MCID computer imaging analysis system. The number of vessels was counted throughout each field of view and was reported as the density of vessels in the IBZ. For measurement of synapse density, a threshold for the synaptophysin immunoreactivity was selected based on the contralateral homologous positive regions by using the “set color threshold” feature in the analysis software. The same threshold was applied to the same set of images that were obtained at equal objectives and light intensities. The data represent the mean area occupied by synaptophysin immunoreactivity, expressed as a percentage of total scan area.

### Data analysis

All data were expressed as mean ± SEM (standard error of mean) unless indicated otherwise. SPECT data were evaluated using repeated measure analysis of variance (ANOVA) followed by post-hoc test Fisher’s PLSD, with p value of ≤0.05 considered statistically significant. All other histological analyses were also evaluated by student t-test and a p-value of ≤0.05 was considered significantly different. The global test using Generalize Estimating Equation (GEE) was implemented to test the group difference on functional recovery measured from three behavioral tests and a p-value of ≤0.05 was considered significantly different.

## Results

### Labeling efficiency and viability (Additional file 1)

When hUTC were labeled in suspension following trypsinization, viability at the end of the labeling procedure was approximately 59-72% and viability did not improve over time (46-51% at 24 hours). Therefore cells were labeled under adherent condition whereby cell viability at the end of the labeling period was higher (about 95%) and remained high at 24 hours (70-72%) under in vitro conditions. Cellular retention of radioactivity was also high in cells that were labeled under adherent condition. With this optimized method, a labeling efficiency of 59.3±2.35% and cell viability of 89.98±3.62% was achieved at the end of the labeling procedure.

### Biodistribution of In-111 only and In-111 labeled hUTC in different organs

Figures 1 and 2 show representative images of an animal that received In-111-labeled hUTC or In-111-oxine alone, respectively. Note the considerable changes in the level of radioactivity in the lungs from day 0 to day 3 in animals that received In-111-labeled hUTC. In contrast, a similar pattern of changes in radioactivity was not seen in animals that received In-111-oxine alone and the level of radioactivity in the lung remained relatively unchanged.

**Figure 3:**
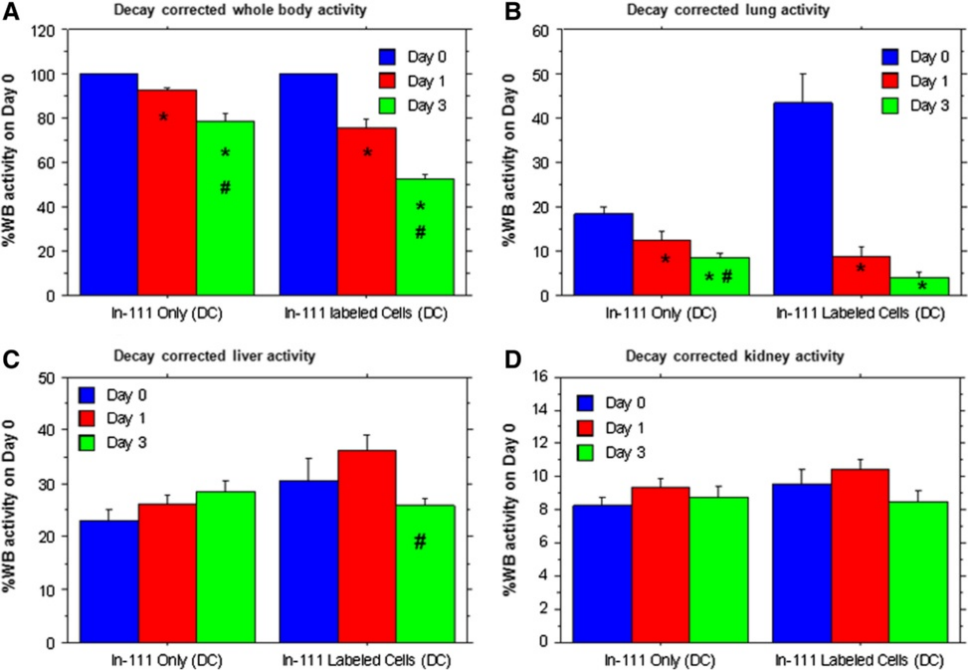
**Biodistribution in the whole body, lungs, liver and kidneys.** Relative percent radioactivity in the whole body (**A**), lungs (**B**), liver (**C**) and kidneys (**D**) of rats that received In-111 oxine vehicle (n=12) or 3 million In-111 labeled hUTC (n=13) 48 hrs after tMCAo. Animals underwent SPECT imaging at day 0, 1 and 3 post-administration and data was normalized to the body radioactivity content on day 0 and corrected for In-111 half-life. WB = whole body, Dc = decay corrected. * = significant differences compared to day 0, # = significant difference between day 1 and 3.

Figure 3 shows the decay corrected percent of administered dose (radioactivity on day 0), present in various organs over time following the administration of In-111-labeled hUTC or In-111 oxine alone (vehicle) to rats. Rapid clearance of radioactivity was observed from the whole body in animals that received In-111-labeled hUTC (Figure 3A). When the percent of administered In-111-labeled hUTC dose was calculated for various organs, most of the radioactivity was observed in the lungs on day 0 following administration. The lung radioactivity on day 0 was 43.36±23.07% of administered dose. On days 1 and 3, the radioactivity in the lungs decreased drastically to 8.81±7.75 and 4.01±4.52%, respectively. In contrast, the animals that received In-111-oxine alone showed 18.38±5.45% of administered dose on day 0 in the lungs, which did not substantially decrease from day 1 (12.59±5.94%) to day 3 (8.34±4.25%) (Figure 3B). The percent-administered dose remained relatively stable in the liver (Figure 3C) for both In-111-labeled hUTC (30.48±14.38, 36.23±9.72, 23.36±8.21% on days 0, 1, and 3, respectively) and In-111 oxine alone (22.89±8.05, 26.11±6.63, 28.32±7.83% on days 0, 1, and 3, respectively). Similarly percent administered dose measured in spleen also remained stable for both In-111-labeled hUTC (5.17±2.37, 6.13±1.09, 4.21±0.57% on days 0, 1, and 3, respectively) and In-111 oxine alone (5.73±2.56, 7.40±1.24, 7.57±1.65% on days 0, 1, and 3, respectively). The percent of administered dose measured in the kidney of rats administered In-111-labeled hUTC was very similar to that observed in animals treated with In-111 only on days 0, 1 and 3 (Figure 3D), suggesting that the kidneys were the route of free In-111 elimination.

**Figure 4:**
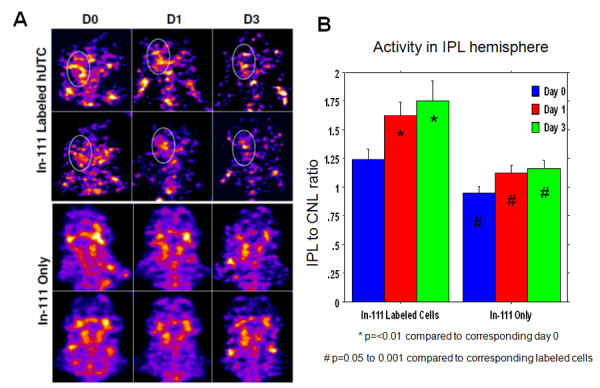
**Accumulation of administered In-111 labeled hUTC in the stroke lesion compared to that of In-111 oxine treated animals.** Images were from two representative animals in respective groups in In-111 labeled hUTC (top panel) and In-111 oxine vehicle (bottom panel). SPECT images showed higher radioactivity (**A**) in the lesions (ipsilateral side (dotted circle) and top panel) and the semi-quantitative analysis showed increased in the ipsilateral to contralateral ratio in animals that received In-111 labeled hUTC (n=13) or In-111 oxine vehicle (n=12) (**B**). * = significant differences compared to corresponding day 0, and # significant differences compared to corresponding In-111-labeled hUTC.

One of the main goals of this study was to determine whether administered hUTC migrate to the sites of stroke (right hemisphere). SPECT scanning was used to determine the accumulation of In-111-labeled hUTC at the site of stroke. Radioactivity in the left hemisphere was considered to be non-specific for all animals; therefore, radioactivity in the right hemisphere (stroke site) was normalized to the radioactivity measured in the left hemisphere (R/L). It is expected that in rats that received In-111 oxine alone, radioactivity would not accumulate preferentially in the site of lesion and that the radioactivity would not be increased compared to contralateral hemisphere (R/L ratio near one) in contrast to animals that received In-111-labeled hUTC. It was hypothesized that animals administered In-111-labeled hUTC would show significantly higher R to L ratio due to specific migration of hUTC to the stroke area. Figure 4A shows representative images of the accumulation of radioactivity to the site of the stroke lesion following the administration of In-111-labeled hUTC to rats. The R/L ratio was significantly higher (p<0.05) on days 0, 1, 3 in animals that received In-111-labeled hUTC (1.24±0.29, 1.63±0.39, 1.75±0.59, respectively) compared to the animals that received In-111 oxine alone (vehicle) (0.95±0.18, 1.13±0.22, 1.16±0.29, respectively) (Figure 4B). The ratio increased significantly (p=<0.01) from day 1 to day 3 in animals that received In-111 labeled hUTC; however, a similar pattern of radioactivity ratio was not observed in animals administered In-111-oxine alone (Figure 4B), indicating specific accumulation of cells at the site of the lesion.

### Neurobehavioral improvement following hUTC administration

Due to loss of animals during 12–13 weeks follow up, data from 11 animals that received In-111-labeled hUTC cells and data from 10 animals that received In-111-oxine only are included. Rats subjected to In-111 labeled hUTC treatment 48 hrs after tMCAo exhibited significant neurobehavioral improvements measured by mNSS, adhesive removal test, and foot-fault test starting at day 14 and persisting to day 90 compared to vehicle (In-111-oxine) treated rats (p<0.05, Generalize Estimating Equation test) (Table 1).

**Table 1:** Results of neurobehavioral testing in animals that received either In-111-oxine or 3 million In-111-labeled hUTC (In-111 hUTC) 48 hours post-tMCAo

	**mNSS score**		**Adhesive removal time score (sec)**		**% Left foot-fault score**	
**Days**	**In-111 oxine**	**In-111 hUTC**	**In-111 oxine**	**In-111 hUTC**	**In-111 oxine**	**In-111 hUTC**
	**Mean±SD(n)**	**Mean±SD(n)**	**Mean±SD(n)**	**Mean±SD(n)**	**Mean±SD(n)**	**Mean±SD(n)**
1	11.2±0.63(10)	11.5±0.52(11)	115.4±5.18(10)	117.0±4.34(11)	36.6±3.24(10)	36.5±3.30(11)
7	―	―	―	―	―	―
14	9.0±0.94(10)	8.5±0.69(11)	104.2±3.83(10)	100.4±6.83(11)	25.5±3.44(10)	24.3±2.94(11)
21	7.7±0.82(10)	6.9±0.54(11)*	91.1±9.88(10)	85.3±9.64(11)*	19.6±3.13(10)	17.4±3.11(11)*
28	7.2±0.79(10)	6.3±0.65(11)*	79.2±6.38(10)	66.0±9.95(11)*	17.9±2.64(10)	15.5±2.62(11)*
35	6.5±0.85(10)	5.5±0.69(11)*	71.1±5.11(10)	58.7±8.10(11)*	15.6±2.01(10)	13.5±1.81(11)*
42	6.2±0.79(10)	5.4±0.50(11)*	50.4±6.27(10)	41.3±9.31(11)*	14.0±2.49(10)	12.0±1.55(11)*
49	6.0±0.67(10)	5.1±0.30(11)*	45.6±6.83(10)	35.6±8.39(11)*	13.0±2.49(10)	11.0±1.34(11)*
56	5.6±0.52(10)	4.9±0.30(11)*	31.9±11.10(10)	21.5±9.25(11)*	12.2±2.39(10)	10.3±1.19(11)*
70	5.6±0.52(10)	4.8±0.60(11)*	78.4±13.76(10)	62.4±13.77(11)*	11.3±1.77(10)	9.9±1.14(11)*
84	5.4±0.52(10)	4.6±0.50(11)*	71.0±14.50(10)	45.2±6.73(11)*	10.9±1.91(10)	8.6±1.12(11)*
90	5.4±0.52(10)	4.6±0.50(11)*	61.3±12.73(10)	35.4±6.43(11)*	10.2±1.55(10)	8.5±0.93(11)*

The dada are expressed in mean±SD. Number of animals are indicated within parenthesis. * = significant differences.

### Histological analysis

**Figure 5:**
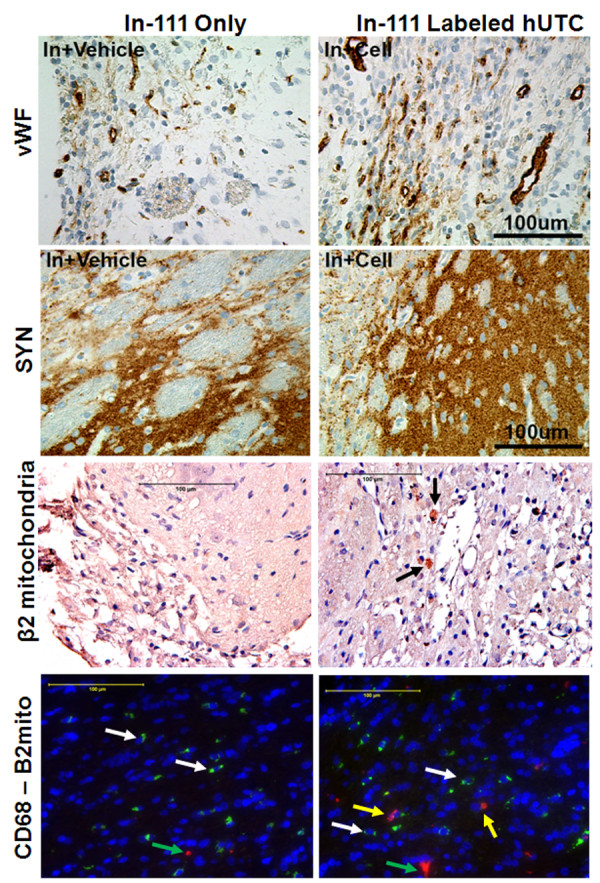
**vWF, Synaptophysin, human cell specific staining (anti β2 mitochondrial antibody) and staining for rat macrophages.** Upper (first) panel: vWF staining showed significantly higher number of vWF positive cells in animals that received In-111 labeled hUTC. 2nd panel: Similarly synaptophysin staining also showed significantly increased expression in animals that received In-111 hUTC. 3rd panel: A few human specific β-mitochondria positive cells were observed in animals that received In-111 hUTC (black arrows) indicating presence of human cells. Lower (fourth) panel: Sections are from IBZ in animals that received In-111-oxine (left) and In-111-labeled hUTC (right). CD68 positive cells (green color, white arrows) are abundant in IBZ. However, a few human cells (Red color, yellow arrows) are seen in animals that received labeled cells. A few red colored spots are seen in both sections (green arrows), which do not correspond to any nucleus (DAPI positive) are probably artifacts.

Table 2 and Figure 5 summarize the histological findings for the two treatment groups. We have previously demonstrated that administration of hUTC effectively improves neurological functional outcome without the reduction of lesion volume [12]. In the present study, lesion volume was evaluated in rats that received 111 labeled hUTC or vehicle (In-111-oxine) 12 weeks post-treatment.

**Table 2:** Histological measurements: Mean ± SD

**Conditions**	**In-111-oxine**	**In-111 labeled hUTC**
% Lesion volume (a)	27.61±14.56 (10)	26.68±15.19 (11)
vWF+ vessels/mm2 (b)	200.45±43.05 (10)	243.90±43.45 (11)*
% Synaptophysin+ area (c)	11.26±1.31 (10)	14.36±4.30 (11)*
TUNEL+ cells/mm2 (d)	35.55±18.08 (10)	20.37±11.55 (11)

N = number of rats/group is indicated in the brackets.

(a) Lesion volume is expressed as a percent compared to the contralateral intact section of the brain.

(b) von Willebrand factor (vWF) is a marker for blood vessels. Density of vWF vessels in IBZ (vessels/mm^2^).

(c) Synaptophysin, a marker of synapses, is expressed as a percent area in the IBZ.

(d) Apoptosis is expressed as the number of apoptotic cells per mm^2^.

Asterisk indicates a significant difference.

No significant differences in the percent lesion volume could be detected after 12 weeks in the brain of rats administered with In-111-labeled hUTC 48 hrs after stroke compared to In-111 oxine alone (Table 2).

### Vascular density in the ischemic boundary zone (IBZ)

The density of blood vessels as monitored by vWF immunoreactivity was significantly higher in the brains of rats that received three million In-111-labeled hUTC compared to In-111 oxine treated group (Figure 5) after 12 weeks following the administration of In-111 labeled hUTC 48 hrs after stroke. These data are consistent with our previous findings [12] demonstrating significantly greater vascularization following the administration of unlabeled hUTC to rats following tMCAo.

### Synaptophysin expression in the IBZ

Synaptophysin, a presynaptic vesicle protein, is used as a marker of synaptogenesis [23]. The expression of synaptophysin was significantly (p<0.05) higher in the IBZ of animals treated at 48 hrs post-tMCAo with three million In-111-labeled hUTC compared to In-111 oxine alone (Figure 5). These data are consistent with our previous findings [12] demonstrating increases in synaptic density following the administration of unlabeled hUTC to rats following tMCAo.

### TUNEL positive cells in the brain

TUNEL histochemistry showed apoptotic cells with typical dark brown, rounded or oval apoptotic bodies (figure not shown). Scattered apoptotic cells were present in the damaged tissue, the majority of which was located in the IBZ. Although no significant differences in the number of TUNEL positive cells in the ipsilateral hemisphere were detected among the two groups, there was a higher number of apoptotic cells present in animals that received In-111 oxine only (35.55±18.08 vs 20.37±11.55, In-111-oxine vs In-111 hUTC).

### Presence of human cells and host macrophages in the IBZ

Figure 5 also shows the presence of β2-mitochondria positive cells in the area of IBZ in animals that received In-111-labeled hUTC. Similar dark brown cells were not observed in animals that received In-111-oxine only. Double stained sections showed clear separation of two populations of cells. Macrophages (CD68+) are seen mostly within the center of stroke as well as at IBZ. There were very few human β2-mitochondria positive cells in the IBZ. CD68+ cells were clearly separated from human cells indicating that hUTC were not engulfed by macrophages.

## Discussion

The findings in the present study have clearly shown the utilization of *in vivo* SPECT imaging for tracking In-111-labeled hUTC to different organs following their intravenous administration. The accumulation of In-111-labeled hUTC and biodistribution differed from that of In-111-oxine alone. The SPECT imaging showed the migration and accumulation of hUTC to the site of stroke lesion in a rat model of tMCAo, which were detected as early as a few hours following IV administration.

### In-111 labeling

Labeling of cells under adherent conditions resulted in higher viability and labeling efficiency. Although a lower labeling efficiency was observed in cells labeled under an adherent condition, long-term cellular viability (at 24 hours) and In-111 retention within cells was higher. The lower cellular retention of In-111 when hUTC were labeled in a suspension conditions was likely related to a significantly higher death rate occurring at 24 hours under the culture conditions. The higher death rate observed in cells labeled in suspension compared to an adherent condition might not be attributed to the amount of In-111-oxine used (a similar dose of In-111-oxine was used in both cases) but was instead related to the lack of proper acclimatization of the cells to the media soon after trypsinization. Similar results were observed when cells were labeled soon after thawing (data not shown). It appears that cells needed acclimatization to the new media for a few hours prior to labeling or needed to be attached before being labeled with In-111 oxine. Although the cellular proliferation studies of In-111 labeled and unlabeled hUTC were not the focus of this study, a transient decrease in the number of cells was observed following labeling with In-111 oxine for up to 3 days (cell viability was not different from that of control and unlabeled cells), however, labeled cells showed a similar increase in cell number between days 3 and 14 compared to that of unlabeled cells (end point of the study) (data not shown). The transient difference in cell numbers might be attributed to the effect of ionizing radiation on the cell cycle [24]. This observation was consistent with the dose response decrease in cell number and viability observed when cells were exposed to increasing amounts of In-111 oxine *in vitro* (Additional file 1) [24,25]. Yoon et al. [24] demonstrated that rat bone marrow mesenchymal stem cells (rMSC) labeled with 1–4 Bq In-111/cell did not show a detrimental effect on the viability and proliferation status of In-111 labeled cells, however, the same investigators reported that In-111, at levels of 4.4 Bq/cell, caused a transient decrease in cellular proliferation with recovery of BrdU uptake by day 7. On the other hand, cells exposed to a higher dose (11.1 Bq/cell) caused permanent cell cycle arrest. The dose used in this current study was 6.17 Bq/cell, which caused transient decrease in cell number with full recovery observed after 3 days of culture consistent with published reports [24]. In addition, other investigators have shown a similar effect of In-111 on the viability and proliferation of CD34+ hematopoietic stem cells (HSC) [26].

### IV administered hUTC, in vivo imaging and advantage of nuclear medicine technique

Investigators have tracked In-111-labeled stem cells and dendritic cells following IV administration in various animal disease models as well as in human clinical studies [27-30]. Most investigations that used In-111-labeled cells were primarily directed to the detection of cells in myocardial infarction in animals or in humans. Investigators also used intra-arterial injection of Tc-99m-labeled bone marrow mononuclear cells in chronic stroke patients to determine the distribution in the stroke areas and in other organs [31,32]. These investigators used autologous bone marrow cells, which were not sorted for specific stem cell lineages and were mixtures of different cell types. Makinen et al. has used In-111-labeled cord blood mononuclear cells (hematopoietic stem cells like cells) in stroke rats and did not observe any migration of cells at the site of stroke [33]. To our knowledge, this is the first study that utilizes *in vivo* SPECT imaging to determine the accumulation of In-111-labeled hUTC to the sites of stroke in a rat model of tMCAo. The primary purpose of these studies was to determine the initial distribution and specific migration of IV administered hUTC to the sites of stroke and other organs. We choose IV over intra-arterial injection to avoid further blocking of small patent vessels in stroke areas (micro thrombo-embolism due to cell masses). The semi-quantitative analyses have shown a differential distribution of In-111 labeled hUTC in the lungs and liver over a 3–day period. The findings are consistent with previous investigations demonstrating that most of the administered cells trafficked to the lungs soon after IV injection and then redistributed to other organs over time [27,33-35]. The pattern of distribution and the percent of administered dose observed in various organs of In-111 labeled hUTC treated animals differ considerably from rats administered In-111 oxine alone. A considerable decrease in the percent of radioactivity was observed in lungs of rats administered In-111 hUTC (from over 40% to 10%) within 24 hours that might be attributed to the release and relocation of hUTC from the lungs to other organs. The relocation of cells from lungs to other organs is supported by data showing an increase in the percent radioactivity detected in the liver on day 1 compare to day 0. Similar findings were also reported by other investigators, where mesenchymal stem cells showed relocation from lungs to the liver and spleen [27,34,35]. The radioactivity seen in the lungs, liver and spleen following administration of In-111-labeled hUTC was thought to be due to the radioactivity associated with the cells (not due to the release of In-111 bound to cellular protein and subsequent labeling of adjacent cells). In-111 oxine (oxyquinoline) complex is neutral and lipid-soluble, which enables it to penetrate cell membrane quickly. Once within the cell In-111 becomes firmly attached to cytoplasmic components, which chelate In-111 more strongly than oxyquinoline. Once In-111 is released as a result of cell lysis, it cannot label surrounding cells because the In-111 complex to cellular protein can no longer penetrate the cellular membrane and therefore is excreted through the kidney (see below). There was a significant difference in the R/L ratios (ipsilateral/contralateral ratios) between the groups of rats that received In-111 labeled hUTC or In-111-oxine alone, indicating the distribution of hUTC to the site of injury and accumulation over time. Although it cannot be ascertained from SPECT imaging whether In-111 was associated with cells, results showed that a small percentage of the signal is observed in the kidney, the proposed route of In-111 elimination, suggesting the presence of “free” In-111. We did not further characterize the specificity of the signal obtained in these animals. In a previous study, a similar time dependent accumulation of neural progenitor cells in stroke areas was observed following intrathecal administration [36], although cellular magnetic resonance imaging (MRI) was applied to detect the neural progenitor cells. In an ongoing investigation, In-111-labeled endothelial progenitor cells show similar time dependent accumulation to the sites of brain tumor (unpublished data). In the current study, *in vivo* imaging showed the specific accumulation and retention of administered hUTC to the sites of stroke, which was also confirmed by the presence of human cells in the stroke areas. Although we have not sacrificed animals at an early time point to confirm the migration of human cells at the site of stroke, early migration of umbilical cord blood derive mesenchymal stem cells to the sites of stroke following IV and intrathecal administration has been demonstrated [37]. Different imaging modalities as well as *in vivo* detection techniques have been used to determine the migration and distribution of IV administered stem cells in various organs or area of interests [38,39]. Our previous studies as well as other investigators have shown that the migration and accumulation of labeled cells to the sites of lesions in various disease models using *in vivo* MRI, however, no data were provided on the tracking and distribution of cells to other organs [13,40]. This is because of large background signal from subject interfaces in other organs and time to compensate loss in SNR (signal to noise ratio) due to less sensitive bigger RF coil in small animal MRI system. Optical imaging systems are capable of imaging whole animals but currently available imaging systems lack the required resolution to identify the depth of incoming signals and the sensitivity necessary to detect a small number of accumulated cells. Attenuation (increased in light scattering with depth) of fluorescent or bioluminescent signals is also an issue. Investigators also failed to report detectable level of human cell markers using rtPCR in various organs of interests following IV administration of cells over time [27,41]. In this respect, nuclear medicine imaging has an advantage over other available techniques being highly sensitive as well as having the option to use large field of view to cover most of the animal body in a single scan and capable to capture dynamic changes in the biodistribution of cells in live animals. The current investigation showed clear differences in the distribution patterns of radioactivity at the sites of stroke and in various organs in rats administered In-111 labeled hUTC as compared to In-111-oxine only.

Significant changes in the radioactivity patterns were observed in the whole body and the lungs. There was also a difference in the percent of the radioactivity uptake in the liver of In-111-labeled hUTC administered group at day 3 compared to the In-111-oxine administered animals. A possible explanation for these changes in cell trafficking was that, following their intravenous administration, the cells first passed through the lungs and liver where they remained entrapped in the capillary systems representing almost 80% of the administered radioactive dose (based on whole body radioactivity on day 0). Then the entrapped cells may be dislodged and recirculate and accumulated to distal sites (such as lesion site, bone marrow or organs of interest). A increase in radioactivity in the spleen on day 1 was also observed in this study. Some entrapped cells could have been lysed. Radioactivity in lungs indicated clearly the initial nonspecific entrapment with rapid clearance, which is in agreement with other investigations [27,34,35]. A negligible “free” In-111 in the circulation was observed following IV administration of In-111 labeled hUTC as demonstrated by the absence of radioactivity in the heart area whereas a strong radioactive signal was observed in animals that received of In-111 oxine only. In addition, the lack of increase in radioactivity signal observed in the kidney at later time points was also indicative of a negligible amount of the presence of “free” In-111. Similar radioactivity signals in the kidneys for both In-111-oxine only and In-111 labeled hUTC groups suggested that the kidney was the route of excretion of In-111 and represented a negligible amount. In the present *in vivo* study also showed the specific accumulation and retention of administered hUTC to the sites of stroke, which was also confirmed by the presence of human cells in the stroke areas. This is in agreement with our previous studies, where the migration of administered hUTC to the sites of stroke was demonstrated by histochemical analyses [12]. Although the ability of nuclear medicine techniques to track administered cells is limited due to the short half-life of usable radiopharmaceuticals for cell labeling, early migration as well as redistribution of stem cells following IV administration can be confidently assessed for at least a few days. Moreover, FDA approved radiopharmaceuticals are readily available for cell labeling and for clinical imaging study with less regulatory hurdles.

### Specificity of cell associated activity vs free In-111

It was observed that a negligible amount of “free” In-111 existed in the circulation following IV administration of In-111 labeled hUTC as demonstrated by the absence of radioactivity in the heart area whereas a strong radioactive signal was observed in animals that received of In-111 oxine only. The strong radioactive signal observed in the heart area of animals that received In-111-oxine alone was probably due to red blood cells (RBC) and plasma protein bound to In-111. Wistow et al. [42] showed that 67% and 33% of radioactive oxine (Tc-99 m-oxine) was bound to RBC and plasma protein, respectively, when whole blood was incubated with Tc-99 m-oxine. The protein and RBC bound to In-111 was very stable and less that 1% excretes through feces and urine when In-111 oxine alone was injected intravenously. Thakur et al. [43] indicated that the efflux of In-111 from labeled cells was very negligible and most of the efflux happened within 5 minutes following the labeling procedure. Moreover, more than 90% of In-111 radioactivity was found to be associated with labeled white blood cells (WBC) in patients 20 hours following intravenous administration of In-111 labeled WBC [44]. Therefore, it can be concluded that the activity seen at the sites of stroke in animals that received In-111 labeled hUTC was due to the radioactivity associated with the cells. It could be argued that transmigration of activity from lungs to the lesions is attributed to the migration of macrophages that had phagocytosed the In-111 labeled hUTC accumulated (impacted) in the capillary processes of lung. Based on our previous experiences, this is a very unlikely scenario. In previous studies we have proven that IV administered stem cells that had migrated to the sites of lesion were clearly distinguished from the host macrophages. None of the human marker positive cells superimposed with host macrophages and vice versa [45,46]. On the other hand, if the released In-111 tagged with albumin and accumulated to the site of stroke, we should have seen higher uptake in animals that received In-111-oxine only. Compared to free In-111, In-111-oxine has more avidity for serum albumin. Double staining for both rat macrophage and human marker in the current study demonstrated that human cells were not engulfed by host macrophages, which is also supported our previous findings [45,46].

### Histological analysis

The primary aim of cell based therapy is to enhance the recovery of function. The relationship between neurological function and the histological response in the brain following stroke warrants investigation. Improvement of functions as well as histochemical changes has been reported following the administration of bone marrow derived stem cells in a rat model of stroke [47]. Similar to a previous report [12], increases in vascular and synaptic densities in the vicinity of the ischemic boundary zone (IBZ) have been reported following the administration of In-111 labeled hUTC, although the lesion size and the number of apoptotic cells did not differ significantly between the two groups of animals. Human cell specific markers showed the presence of human cells in the vicinity of the stroke in animals that received In-111 labeled hUTC. With the added small amount of In-111 radioactivity, we did not expect a reduction in the efficacy of hUTC as demonstrated by the behavioral improvement such as modified neurological severity score (mNSS), adhesive removal tests and foot fault measurement and increases in markers of vascularization and synaptic plasticity, consistent with the previously reported study on the efficacy of hUTC on restorative events [12]. These improvements of vascular and synaptic densities may be due to the bystander hUTC as well as paracrine effects of the cells that have been reported by others as well as by our group [7,12,47]. We have not used immunosuppressant to reduce host’s response to administered foreign cells. However, it has been noted by different investigators that stromal cells as well as umbilical tissue derived cells that express MHC I molecules but show absence of MHC II molecular are immunomodulatory and can be used in allogeneic transplantation [48-50]. Histochemical analysis showing separation of human marker positive and host macrophage marker positive cells also indicated the absence of graft vs host reaction.

## Conclusion

The present study demonstrated a useful technique to label hUTC with In-111-oxine. It was demonstrated the presence of signal from In-111 labeled hUTC to the site of stroke in a rat model of tMCAo using *in vivo* SPECT imaging. Animals treated with In-111 labeled hUTC showed improvement at the cellular level in the IBZ as indicated by the increased vascularization and synaptic density in the treated tissues. In-111-oxine labeling is safe and the labeling does not seem to have any detrimental effect on cell viability and therapeutic effects.

## Competing interests

The authors declare that they have no competing interests.

## Authors' contributions

ASA carried out the experimental design, optimization of labeling of cells, data analysis and manuscript writing. CT participated in experimental design, cell labeling, data analysis, and manuscript writing. BN, SJV, and KH supplied the cells, participated in experimental design and logistics, and manuscript writing. LZ and QJ made the model, participated in histochemistry data analysis, conducted neurobehavioral assay, NRSV and AI maintained the animals, prepared the stem cells for injection, and acquired the SPECT images. MC participated in overall project management. All authors read and approved the final manuscript.

## Disclaimer

Klaudyne Hong, PhD, Stroke Team Leader is an employee of ATRM. Stephen Victor, MD, is a Senior Medical Director at Janssen Research and Development. Christine Thiffault, PhD, and Bradford Navia, MD, were employees of ATRM and Janssen, respectively, at the time that the study was conducted.

## Funding

The research presented herein was funded by Advanced Technologies and Regenerative Medicine, LLC.

## Pre-publication history

The pre-publication history for this paper can be accessed here:

http://www.biomedcentral.com/1471-2342/12/33/prepub

## Supplementary Material

Additional file 1 Optimization of hUTC labeling procedures using In-111-oxine. Click here for file

## References

[B1] Lloyd-Jones D Adams R Carnethon M De Simone G Ferguson TB Flegal K Ford E Furie K Go A Greenlund K Haase N Hailpern S Ho M Howard V Kissela B Kittner S Lackland D Lisabeth L Marelli A McDermott M Meigs J Mozaffarian D Nichol G O'Donnell C Roger V Rosamond W Sacco R Sorlie P Stafford R Steinberger J Heart disease and stroke statistics–2009 update: a report from the American Heart Association Statistics Committee and Stroke Statistics Subcommittee Circulation 2009 119 3 480 486 19171871 10.1161/CIRCULATIONAHA.108.191259

[B2] Roger VL Go AS Lloyd-Jones DM Benjamin EJ Berry JD Borden WB Bravata DM Dai S Ford ES Fox CS Fullerton HJ Gillespie C Hailpern SM Heit JA Howard VJ Kissela BM Kittner SJ Lackland DT Lichtman JH Lisabeth LD Makuc DM Marcus GM Marelli A Matchar DB Moy CS Mozaffarian D Mussolino ME Nichol G Paynter NP Soliman EZ Heart disease and stroke statistics–2012 update: a report from the American Heart Association Circulation 2012 125 1 e2 e220 22179539 10.1161/CIR.0b013e31823ac046 PMC4440543

[B3] Haley EC Jr Brott TG Sheppard GL Barsan W Broderick J Marler JR Kongable GL Spilker J Massey S Hansen CA Pilot randomized trial of tissue plasminogen activator in acute ischemic stroke. The TPA Bridging Study Group Stroke 1993 24 7 1000 1004 10.1161/01.STR.24.7.1000 8322373

[B4] Sen S Huang DY Akhavan O Wilson S Verro P Solander S IV vs. IA TPA in acute ischemic stroke with CT angiographic evidence of major vessel occlusion: a feasibility study Neurocrit Care 2009 11 1 76 81 10.1007/s12028-009-9204-1 19277904

[B5] Cao FJ Feng SQ Human umbilical cord mesenchymal stem cells and the treatment of spinal cord injury Chin Med J (Engl) 2009 122 2 225 231 19187651

[B6] Secco M Moreira YB Zucconi E Vieira NM Jazedje T Muotri AR Okamoto OK Verjovski-Almeida S Zatz M Gene expression profile of mesenchymal stem cells from paired umbilical cord units: cord is different from blood Stem Cell Rev 2009 5 4 387 401 10.1007/s12015-009-9098-5 20058202 PMC2803263

[B7] Fan CG Zhang QJ Zhou JR Therapeutic potentials of mesenchymal stem cells derived from human umbilical cord Stem Cell Rev 2011 7 1 195 207 10.1007/s12015-010-9168-8 20676943

[B8] Boldicke T Tesar M Griesel C Rohde M Grone HJ Waltenberger J Kollet O Lapidot T Yayon A Weich H Anti-VEGFR-2 scFvs for cell isolation. Single-chain antibodies recognizing the human vascular endothelial growth factor receptor-2 (VEGFR-2/flk-1) on the surface of primary endothelial cells and preselected CD34+ cells from cord blood Stem Cells 2001 19 1 24 36 10.1634/stemcells.19-1-24 11209088

[B9] Okada H Nagamura-Inoue T Mori Y Takahashi TA Expansion of Valpha24(+)Vbeta11(+) NKT cells from cord blood mononuclear cells using IL-15, IL-7 and Flt3-L depends on monocytes Eur J Immunol 2006 36 1 236 244 10.1002/eji.200526085 16380959

[B10] Janic B Guo AM Iskander AS Varma NR Scicli AG Arbab AS Human cord blood-derived AC133+ progenitor cells preserve endothelial progenitor characteristics after long term in vitro expansion PLoS One 2010 5 2 e9173 10.1371/journal.pone.0009173 20161785 PMC2820083

[B11] Lund RD Wang S Lu B Girman S Holmes T Sauve Y Messina DJ Harris IR Kihm AJ Harmon AM Chin FY Gosiewska A Mistry SK Cells isolated from umbilical cord tissue rescue photoreceptors and visual functions in a rodent model of retinal disease Stem Cells 2007 25 3 602 611 17053209 10.1634/stemcells.2006-0308

[B12] Zhang L Li Y Zhang C Chopp M Gosiewska A Hong K Delayed administration of human umbilical tissue-derived cells improved neurological functional recovery in a rodent model of focal ischemia Stroke 2011 42 5 1437 1444 10.1161/STROKEAHA.110.593129 21493915

[B13] Arbab AS Yocum GT Kalish H Jordan EK Anderson SA Khakoo AY Read EJ Frank JA Efficient magnetic cell labeling with protamine sulfate complexed to ferumoxides for cellular MRI Blood 2004 104 4 1217 1223 10.1182/blood-2004-02-0655 15100158

[B14] Janic B Rad AM Jordan EK Iskander AS Ali MM Varma NR Frank JA Arbab AS Optimization and validation of FePro cell labeling method PLoS One 2009 4 6 e5873 10.1371/journal.pone.0005873 19517015 PMC2690694

[B15] Chen J Li Y Wang L Zhang Z Lu D Lu M Chopp M Therapeutic benefit of intravenous administration of bone marrow stromal cells after cerebral ischemia in rats Stroke 2001 32 4 1005 1011 10.1161/01.STR.32.4.1005 11283404

[B16] Schallert T Kozlowski DA Humm JL Cocke RR Use-dependent structural events in recovery of function Adv Neurol 1997 73 229 238 8959217

[B17] Zhang L Schallert T Zhang ZG Jiang Q Arniego P Li Q Lu M Chopp M A test for detecting long-term sensorimotor dysfunction in the mouse after focal cerebral ischemia J Neurosci Methods 2002 117 2 207 214 10.1016/S0165-0270(02)00114-0 12100987

[B18] Jiang Q Thiffault C Kramer BC Ding GL Zhang L Nejad-Davarani SP Li L Arbab AS Lu M Navia B Victor SJ Hong K Li QJ Wang SY Li Y Chopp M MRI detects brain reorganization after human umbilical tissue-derived cells (hUTC) treatment of stroke in rat PLoS One 2012 7 8 e42845 10.1371/journal.pone.0042845 22900057 PMC3416784

[B19] Swanson RA Morton MT Tsao-Wu G Savalos RA Davidson C Sharp FR A semiautomated method for measuring brain infarct volume J Cereb Blood Flow Metab 1990 10 2 290 293 10.1038/jcbfm.1990.47 1689322

[B20] Swana GT Swana MR Bottazzo GF Doniach D A human-specific mitochondrial antibody its importance in the identification of organ-specific reactions Clin Exp Immunol 1977 28 3 517 525 330060 PMC1541017

[B21] Moelker AD Baks T Wever KM Spitskovsky D Wielopolski PA van Beusekom HM van Geuns RJ Wnendt S Duncker DJ van der Giessen WJ Intracoronary delivery of umbilical cord blood derived unrestricted somatic stem cells is not suitable to improve LV function after myocardial infarction in swine J Mol Cell Cardiol 2007 42 4 735 745 10.1016/j.yjmcc.2007.01.005 17320899

[B22] Pillekamp F Reppel M Rubenchyk O Pfannkuche K Matzkies M Bloch W Sreeram N Brockmeier K Hescheler J Force measurements of human embryonic stem cell-derived cardiomyocytes in an in vitro transplantation model Stem Cells 2007 25 1 174 180 10.1634/stemcells.2006-0094 16973834

[B23] Stroemer RP Kent TA Hulsebosch CE Neocortical neural sprouting, synaptogenesis, and behavioral recovery after neocortical infarction in rats Stroke 1995 26 11 2135 2144 10.1161/01.STR.26.11.2135 7482662

[B24] Yoon J-K Park B-N Shim W Ahn YH Lee G Transient cell cycle arrest of rat bone marrow mesenchymal stem cells by In-111 labeling J Nucl Med Meeting Abstracts 2010 51 2_MeetingAbstracts 1774-

[B25] Gholamrezanezhad A Mirpour S Ardekani JM Bagheri M Alimoghadam K Yarmand S Malekzadeh R Cytotoxicity of 111In-oxine on mesenchymal stem cells: a time-dependent adverse effect Nucl Med Commun 2009 30 3 210 216 210.1097/MNM.1090b1013e328318b328328 10.1097/MNM.0b013e328318b328 19262283

[B26] Brenner W Aicher A Eckey T Massoudi S Zuhayra M Koehl U Heeschen C Kampen WU Zeiher AM Dimmeler S Henze E 111In-labeled CD34+ hematopoietic progenitor cells in a rat myocardial infarction model J Nucl Med 2004 45 3 512 518 15001696

[B27] Kraitchman DL Tatsumi M Gilson WD Ishimori T Kedziorek D Walczak P Segars WP Chen HH Fritzges D Izbudak I Young RG Marcelino M Pittenger MF Solaiyappan M Boston RC Tsui BM Wahl RL Bulte JW Dynamic imaging of allogeneic mesenchymal stem cells trafficking to myocardial infarction Circulation 2005 112 10 1451 1461 Epub 2005 Aug 1429 10.1161/CIRCULATIONAHA.105.537480 16129797 PMC1456731

[B28] Hofmann M Wollert KC Meyer GP Menke A Arseniev L Hertenstein B Ganser A Knapp WH Drexler H Monitoring of bone marrow cell homing into the infarcted human myocardium Circulation 2005 111 17 2198 2202 10.1161/01.CIR.0000163546.27639.AA 15851598

[B29] Caveliers V De Keulenaer G Everaert H Van Riet I Van Camp G Verheye S Roland J Schoors D Franken PR Schots R In vivo visualization of 111In labeled CD133+ peripheral blood stem cells after intracoronary administration in patients with chronic ischemic heart disease Q J Nucl Med Mol Imaging 2007 51 1 61 66 17372574

[B30] Morse MA Coleman RE Akabani G Niehaus N Coleman D Lyerly HK Migration of human dendritic cells after injection in patients with metastatic malignancies Cancer Res 1999 59 1 56 58 9892184

[B31] da Fonseca LM B Gutfilen B de Castro PH R Battistella V Goldenberg RC Kasai-Brunswick T Chagas CL Wajnberg E Maiolino A Salles Xavier S Andre C Mendez-Otero R de Freitas GR Migration and homing of bone-marrow mononuclear cells in chronic ischemic stroke after intra-arterial injection Exp Neurol 2010 221 1 122 128 10.1016/j.expneurol.2009.10.010 19853605

[B32] da Fonseca LM B Battistella V de Freitas GR Gutfilen B Dos Santos Goldenberg RC Maiolino A Wajnberg E de Castro PH R Mendez-Otero R Andre C Early tissue distribution of bone marrow mononuclear cells after intra-arterial delivery in a patient with chronic stroke Circulation 2009 120 6 539 541 10.1161/CIRCULATIONAHA.109.863084 19667245

[B33] Mäkinen S Kekarainen T Nystedt J Liimatainen T Huhtala T Närvänen A Laine J Jolkkonen J Human umbilical cord blood cells do not improve sensorimotor or cognitive outcome following transient middle cerebral artery occlusion in rats Brain Res 2006 1123 1 207 215 10.1016/j.brainres.2006.09.056 17070789

[B34] Chin BB Nakamoto Y Bulte JW Pittenger MF Wahl R Kraitchman DL 111In oxine labelled mesenchymal stem cell SPECT after intravenous administration in myocardial infarction Nucl Med Commun 2003 24 11 1149 1154 10.1097/00006231-200311000-00005 14569169

[B35] Yoon JK Park BN Shim WY Shin JY Lee G Ahn YH In vivo tracking of 111In-labeled bone marrow mesenchymal stem cells in acute brain trauma model Nucl Med Biol 2010 37 3 381 388 10.1016/j.nucmedbio.2009.12.001 20346878

[B36] Jiang Q Zhang ZG Ding GL Zhang L Ewing JR Wang L Zhang R Li L Lu M Meng H Arbab AS Hu J Li QJ Pourabdollah Nejad DS Athiraman H Chopp M Investigation of neural progenitor cell induced angiogenesis after embolic stroke in rat using MRI NeuroImage 2005 28 3 698 707 10.1016/j.neuroimage.2005.06.063 16112879

[B37] Lim JY Jeong CH Jun JA Kim SM Ryu CH Hou Y Oh W Chang JW Jeun SS Therapeutic effects of human umbilical cord blood-derived mesenchymal stem cells after intrathecal administration by lumbar puncture in a rat model of cerebral ischemia Stem Cell Res Ther 2011 2 5 38 10.1186/scrt79 21939558 PMC3308035

[B38] Rad AM Iskander AS Janic B Knight RA Arbab AS Soltanian-Zadeh H AC133+ progenitor cells as gene delivery vehicle and cellular probe in subcutaneous tumor models: a preliminary study BMC Biotechnol 2009 9 28 10.1186/1472-6750-9-28 19327159 PMC2669076

[B39] Arbab AS Janic B Haller J Pawelczyk E Liu W Frank JA In vivo cellular imaging for translational medical research Curr Med Imaging Rev 2009 5 1 19 38 10.2174/157340509787354697 19768136 PMC2746660

[B40] Anderson SA Glod J Arbab AS Noel M Ashari P Fine HA Frank JA Noninvasive MR imaging of magnetically labeled stem cells to directly identify neovasculature in a glioma model Blood 2005 105 1 420 425 10.1182/blood-2004-06-2222 15331444

[B41] Chapel A Bertho JM Bensidhoum M Fouillard L Young RG Frick J Demarquay C Cuvelier F Mathieu E Trompier F Dudoignon N Germain C Mazurier C Aigueperse J Borneman J Gorin NC Gourmelon P Thierry D Mesenchymal stem cells home to injured tissues when co-infused with hematopoietic cells to treat a radiation-induced multi-organ failure syndrome J Gene Med 2003 5 12 1028 1038 10.1002/jgm.452 14661178

[B42] Wistow BW Grossman ZD McAfee JG Subramanian G Henderson RW Roskopf ML Labeling of platelets with oxine complexes of Tc-99 m and In-111. Part 1. In vitro studies and survival in the rabbit J Nucl Med 1978 19 5 483 487 417153

[B43] Thakur ML Segal AW Louis L Welch MJ Hopkins J Peters TJ Indium-111-labeled cellular blood components: mechanism of labeling and intracellular location in human neutrophils J Nucl Med 1977 18 10 1022 1026 409746

[B44] Thakur ML Lavender JP Arnot RN Silvester DJ Segal AW Indium-111-labeled autologous leukocytes in man J Nucl Med 1977 18 10 1014 1021 409745

[B45] Arbab AS Pandit SD Anderson SA Yocum GT Bur M Frenkel V Khuu HM Read EJ Frank JA Magnetic resonance imaging and confocal microscopy studies of magnetically labeled endothelial progenitor cells trafficking to sites of tumor angiogenesis Stem Cells 2006 24 3 671 678 10.1634/stemcells.2005-0017 16179427

[B46] Arbab AS Janic B Knight RA Anderson SA Pawelczyk E Rad AM Read EJ Pandit SD Frank JA Detection of migration of locally implanted AC133+ stem cells by cellular magnetic resonance imaging with histological findings FASEB J 2008 22 9 3234 3246 10.1096/fj.07-105676 18556461 PMC2518252

[B47] Liu Z Li Y Zhang RL Cui Y Chopp M Bone marrow stromal cells promote skilled motor recovery and enhance contralesional axonal connections after ischemic stroke in adult mice Stroke 2011 42 3 740 744 10.1161/STROKEAHA.110.607226 21307396 PMC3060040

[B48] Zhou C Yang B Tian Y Jiao H Zheng W Wang J Guan F Immunomodulatory effect of human umbilical cord Wharton's jelly-derived mesenchymal stem cells on lymphocytes Cell Immunol 2011 272 1 33 38 10.1016/j.cellimm.2011.09.010 22004796 PMC3235326

[B49] Wang M Yang Y Yang D Luo F Liang W Guo S Xu J The immunomodulatory activity of human umbilical cord blood-derived mesenchymal stem cells in vitro Immunology 2009 126 2 220 232 10.1111/j.1365-2567.2008.02891.x 18624725 PMC2632684

[B50] Yoo KH Jang IK Lee MW Kim HE Yang MS Eom Y Lee JE Kim YJ Yang SK Jung HL Sung KW Kim CW Koo HH Comparison of immunomodulatory properties of mesenchymal stem cells derived from adult human tissues Cell Immunol 2009 259 2 150 156 10.1016/j.cellimm.2009.06.010 19608159

