# Supplementary material for: Tracking of In-111-labeled human umbilical tissue-derived cells (hUTC) in a rat model of cerebral ischemia using SPECT imaging
Source: BMC Med Imaging. 2012 Dec 6;12:33. doi: 10.1186/1471-2342-12-33 (PMC3538050; doi:10.1186/1471-2342-12-33)
Supplement: Additional file 1 — Optimization of hUTC labeling procedures using In-111-oxine. [file 1471-2342-12-33-S1.doc]

**Title:** Tracking of In-111-labeled human umbilical tissue-derived cells (hUTC) in a rat model of cerebral ischemia using SPECT Imaging

**Preparation of cells for labeling with In-111-oxine:** hUTC were kept in 3 ml vials containing 30 million cells in liquid nitrogen until the In-111 labeling procedure. Cells were rapidly thawed in a water bath at 37ºC and placed into a 15 ml conical tube containing 7 ml serum free media and mixed thoroughly by inverting the tube 3-4 times. The cell suspension was then centrifuged at 1000-1200 rpm (173-250 X g) for 5 min at room temperature. Supernatant was removed and the cell pellet was resuspended in 30 ml of complete media (DMEM containing 15% FBS) and the viability was determined using the Trypan blue exclusion method. Six millions viable cells in 15 ml of media were plated onto a T75 flask and incubated for 48 hrs in a humidified and 5% CO_2_ environment at 37ºC before the labeling procedure. More than 90% cells were attached to the surface of the flask by 48 hrs.

**Optimization of the labeling procedures of hUTC with In-111 oxine:** The cells were labeled either in suspension or under adherent condition (T75 flask) and the cellular retention of In-111 was determined in both, serum and serum free conditions to determine whether labeling efficiency, viability, and the cellular retention of In-111 label was dependent on the presence of serum following the labeling procedure. Cell viability and cellular retention of In-111 radioactivity within the cells was determined immediately after labeling (T=0), 2 and 24 hrs post-labeling. Cell viability was determined by Trypan blue dye exclusion method.

a) **hUTC labeled in a suspension condition*:*** Cells were cultured for 48 hours, detached using TryplE (Invitrogen, Carlsbad CA) and washed 3 times with PBS containing Ca^2+^/Mg^2+^. Cells from two T75 flasks (approximately 6 million each) were combined and labeled with 1 mCi of In-111 oxine in a final volume of 2 ml PBS (containing Ca^2+^/Mg^2+^) in a 15 ml conical tube. After 15-20 min incubation at room temperature, cells were washed 3 times with PBS (containing Ca^2+^/Mg^2+^) and were resuspended in either serum containing or serum free media. All supernatant were collected and combined to calculate the labeling efficiency (pls see below). Radioactivity associated with the cells pellet (bound to cells) and supernatant was measured using a dose calibrator (CRC 127R, Capintec Inc., Ramsey NJ). Approximately 1 million cells (in 0.5 ml of either serum containing or serum free media) were re-plated onto a 24-well plate, and cellular retention of In-111 as well as cell viability was determined immediately after labeling (T=0), 2 and 24 hrs post-labeling.

b) **hUTC labeled under adherent condition**: Cells were cultured in flasks for 48 hrs, washed twice with 5 ml PBS (Ca^2+^/Mg^2+^) and labeled with 1 mCi In-111 oxine in 3 ml PBS (Ca^2+^/Mg^2+^) at room temperature for 15-20 minutes. Supernatant was removed (see calculation of labeling efficiency) and cells were washed twice with 5 ml PBS (Ca^2+^/Mg^2+^) and the supernatants were combined with the above. Cells were detached using TryplE and resuspended in either serum containing or serum free media. Radioactivity associated with cell pellet and supernatant was measured using a dose calibrator. Approximately 1 million cells (in 0.5 ml of either serum containing or serum free media) were re-plated onto a 24-well plate, and cellular retention of In-111 as well as cell viability was determined immediately after labeling (T=0), 2 and 24 hrs post-labeling.

**Results:** Table 1 shows cellular viability and In-111 retention over time of hUTC labeled with In-111 oxine in a suspension condition. Results showed that 28-41% of cells died when hUTC were labeled with In-111 oxine in a suspension condition and viability continued to deteriorate over time (49-54% at 24 hrs). The cellular retention of In-111 was moderate in cells labeled in suspension condition (67-74%) after 24 hrs. The presence of serum added after the labeling procedure did not appear to influence the percent In-111 retention overtime but rather prevented cell death.

Although the labeling efficiency was low when cells were labeled under adherent conditions compared to that of cells labeled in suspension, viability was high immediately after labeling (only 5% of cells died after the labeling procedure) and remained at 70-72% after 24 hrs. Leakage of In-111 from the cells was low after 24 hrs (81-82% retention) incubation at 37^o^C. The addition of serum to the cells following 111-In labeling procedure had no effect on In-111 retention or cell viability.

Table 1 shows the viability and In-111 retention over time.

| Labeling Conditions/Retention Conditions | Pre Labeling Viability | % Labeling Efficiency** | Post labeling viability | 2 hr %In-111 retention | 2 hr viability | 24 hrs %In-111 retention | 24 hrs viability |
| --- | --- | --- | --- | --- | --- | --- | --- |
| Suspension/Serum Free | 92.5% | 76.0% | 72.0% | 70.61% | 71.0% | 66.65% | 45.8% |
| Suspension/Serum | 96.8% | 72.9% | 58.4% | 80.70% | 54.1% | 73.89% | 50.6% |
| Adherent/Serum Free | NA | 61.2% | 94.7% | NA | NA | 82.34% | 71.2% |
| Adherent/Serum | NA | 61.2% | 94.7% | NA | NA | 81.19% | 69.4% |

*Average of 2-4 independent experiments

** Labeling efficiency was calculated as follow: (Activity in the cell suspension÷ total activity (cell suspension and combined washes)) X 100 = percent yield,

NA = Not available

Based on the results from the above studies, the following optimization studies were performed to confirm the labeling procedures;

1. ***Labeling of hUTC with In-111 oxine using an optimized method.*** Media was discarded and cells were washed twice with 5 ml serum free media. 1 mCi of In-111 in a total volume of 2.5 ml serum free media (SFM) was added to the flask and incubated at room temperature for either 20 or 30 min. The radioactive SFM was collected after incubation and cells were washed twice with 5 ml PBS. The radioactive supernatants were collected and combined to determine the radioactive yield (see below). Cells were detached using 3 ml TryplE and neutralize with 7 ml of media containing serum. Cells were centrifuged at 1000-1200 rpm (173-250 X g) and the radioactive supernatant was collected and combined with the above. In-111 labeled hUTC were resuspended in 3 ml of media containing serum and viability was determined using the Trypan blue exclusion method. Labeling efficiency was calculated as follow

Radioactivity in the cell suspension X 100 = percent yield

Total radioactivity (cell suspension and combined washes)

About 1 million cells/0.5 ml were re-plated in 24-well low adherence plate (N=6 wells per condition, 20 min vs 30 min incubation). Cellular retention of In-111 radioactivity and viability were determined immediately after labeling (T=0), 2 and 24 hrs.

1. hUTC were subjected to the same labeling procedure as above with the exception that In-111 oxine was omitted from the procedure and serve as a control.

**Results:** Table 2 shows the results of initial labeling and retention studies.

| **Conditions** | % Labeling Efficiency** | Post labeling viability | 2 hr %In-111 retention | 2 hr viability | 24 hrs %In-111 retention | 24 hrs viability |
| --- | --- | --- | --- | --- | --- | --- |
| 30 min incubation | 58% | 94.4% | 92% | 90.12% | 83.5% | 71.34% |
| 20 min incubation | 52.34% | 95.35% | 95.37% | 92.08% | 89.27% | 77.4% |
| Control | NA | 90.39% | NA | 85.29% | NA | 57.25% |

*Average of 2-4 independent experiments

** Labeling efficiency was calculated as follow: (Activity in the cell suspension÷ total activity (cell suspension and combined washes)) X 100 = percent yield,

NA = Not available

Based on the results obtained from the above optimized method, it was determined that the optimum incubation time of hUTC with In-111-oxine was 20-25 min under adherent condition.
